# An unexpectedly high degree of specialization and a widespread involvement in sterol metabolism among the *C. elegans *putative aminophospholipid translocases

**DOI:** 10.1186/1471-213X-8-96

**Published:** 2008-10-02

**Authors:** Nicholas N Lyssenko, Yana Miteva, Simon Gilroy, Wendy Hanna-Rose, Robert A Schlegel

**Affiliations:** 1 Department of Biochemistry and Molecular Biology, The Pennsylvania State University, University Park, Pennsylvania, 16802, USA; 2 Department of Biology, The Pennsylvania State University, University Park, Pennsylvania, 16802, USA; 3 The Huck Institutes of the Life Sciences at the Pennsylvania State University, University Park, Pennsylvania, 16802, USA; 4 Department of Cell Biology, NC10, Lerner Research Institute, 9500 Euclid Avenue, Cleveland Clinic Foundation, Cleveland, Ohio, 44195, USA; 5 Department of Physiology, University of Pennsylvania, Philadelphia, Pennsylvania, 19104, USA; 6 Department of Botany, University of Wisconsin, B117 Birge Hall, Madison, Wisconsin, 53706, USA

## Abstract

**Background:**

P-type ATPases in subfamily IV are exclusively eukaryotic transmembrane proteins that have been proposed to directly translocate the aminophospholipids phosphatidylserine and phosphatidylethanolamine from the exofacial to the cytofacial monolayer of the plasma membrane. Eukaryotic genomes contain many genes encoding members of this subfamily. At present it is unclear why there are so many genes of this kind per organism or what individual roles these genes perform in organism development.

**Results:**

We have systematically investigated expression and developmental function of the six, *tat-1 *through *6*, subfamily IV P-type ATPase genes encoded in the *Caenorhabditis elegans *genome. *tat-5 *is the only ubiquitously-expressed essential gene in the group. *tat-6 *is a poorly-transcribed recent duplicate of *tat-5*. *tat-2 *through *4 *exhibit tissue-specific developmentally-regulated expression patterns. Strong expression of both *tat-2 *and *tat-4 *occurs in the intestine and certain other cells of the alimentary system. The two are also expressed in the uterus, during spermatogenesis and in the fully-formed spermatheca. *tat-2 *alone is expressed in the pharyngeal gland cells, the excretory system and a few cells of the developing vulva. The expression pattern of *tat-3 *is almost completely different from those of *tat-2 *and *tat-4*. *tat-3 *expression is detectable in the steroidogenic tissues: the hypodermis and the XXX cells, as well as in most cells of the pharynx (except gland), various tissues of the reproductive system (except uterus and spermatheca) and seam cells. Deletion of *tat-1 *through *4 *individually interferes little or not at all with the regular progression of organism growth and development under normal conditions. However, *tat-2 *through *4 *become essential for reproductive growth during sterol starvation.

**Conclusion:**

*tat-5 *likely encodes a housekeeping protein that performs the proposed aminophospholipid translocase function routinely. Although individually dispensable, *tat-1 *through *4 *seem to be at most only partly redundant. Expression patterns and the sterol deprivation hypersensitivity deletion phenotype of *tat-2 *through *4 *suggest that these genes carry out subtle metabolic functions, such as fine-tuning sterol metabolism in digestive or steroidogenic tissues. These findings uncover an unexpectedly high degree of specialization and a widespread involvement in sterol metabolism among the genes encoding the putative aminophospholipid translocases.

## Background

Subfamily IV of the P-type ATPase superfamily is a group of exclusively eukaryotic large multipass transmembrane proteins that appear to function as inward – from the exofacial to the cytofacial monolayer – translocases of the aminophospholipids phosphatidylserine (PS) and phosphatidylethanolamine (PE) and of the choline lipid phosphatidylcholine (PC) [1,2]. PS, PE and PC are rather unexpected substrates for these proteins because the "classical" P-type ATPases in subfamilies I, II and III pump metal cations and protons [3]. Two lines of evidence suggest that members of subfamily IV translocate aminophospholipids. First, biochemical investigations have determined that heterologously expressed and purified subfamily IV P-type ATPases progress through the catalytic cycle and hydrolyze ATP in the presence of specifically PS and PE [4,5]. And second, genetic studies have consistently found that deletion of genes encoding subfamily IV members increases exposure on the cell surface of endogenous PS and PE, which are normally mostly concealed, and diminishes translocation to the cytofacial leaflet of exogenously-introduced labeled PS and PE, which are normally quickly internalized [6-10]. Evidence for PC as a transported substrate is much less extensive, consisting of observations of labeled PC internalization in *Saccharomyces cerevisiae *[10]. There is still some skepticism that subfamily IV P-type ATPases directly translocate the three phospholipids (as opposed to pumping some other substance whose concentration gradient drives phospholipid flip by the actual translocase) [11] or that they are directly responsible for internalization of PS, PE and PC (as opposed to facilitating vesicular traffic required for proper working of the actual translocase) [12]. However, neither an alternative candidate transported substrate for these ATPases nor strong evidence for altered vesicular traffic as the cause of PS, PE and PC exposure on the cell surface in subfamily IV P-type ATPase mutants has thus far emerged.

Eukaryotic genomes contain many genes encoding P-type ATPases in subfamily IV (14 in mice and humans) [13]. This brings up the questions: why do eukaryotes require so many putative aminophospholipid translocases, what are the individual functions of these genes, and how should these genes be divided into subgroups in order to study them? Investigations in the single-cell fungus *S*. *cerevisiae *offer some answers. The *S. cerevisiae *genome includes five subfamily IV P-type ATPase genes. One of these, *NEO1*, is essential [14]. The remaining four – *DRS2*, *DNF1*, *DNF2 *and *DNF3 *– are individually dispensable but together comprise an essential subgroup [15]. Although Drs2p resides predominantly in the Golgi apparatus [16] and Dnf1p and Dnf2p reside predominantly in the plasma membrane [10], all three must be deleted for the highest levels of PS and PE exposure on the cell surface [10,12]. These findings imply that a number of subfamily IV P-type ATPases must work in concert to efficiently sequester the two aminophospholipids in the cytofacial leaflet [17]. When extrapolated to multicellular organisms, in which loss of aminophospholipid transmembrane asymmetry and PS appearance on the cell surface are fatal [18], the yeast paradigm predicts that each somatic cell must express at least two individually nonessential but ubiquitous P-type ATPases in subfamily IV: one for the Golgi apparatus and one for the plasma membrane.

We have systematically investigated the six, *tat-1 *through *6*, P-type ATPase subfamily IV genes expressed in the multicellular organism *Caenorhabditis elegans *and found that expression patterns and deletion phenotypes of these genes are inconsistent with the predictions of the yeast paradigm. This does not mean that *C. elegans *P-type ATPases in subfamily IV do not translocate PS and PE. In fact, a recent report shows that loss of *tat-1 *leads to the appearance of PS on the surface of germline and certain somatic cells [6]. Rather, our findings suggest that individually nonessential subfamily IV members, *tat-1 *through *4*, could not accomplish the bulk of aminophospholipid internalization even together as a group and, instead, are specialized to particular tissues, where three of these genes subtly regulate sterol metabolism by, perhaps, adjusting transbilayer lipid distribution. The housekeeping aminophospholipid translocase seems to be encoded by *tat-5*, a homolog of *NEO1*.

## Results

### *C. elegans *animals express six subfamily IV P-type ATPases

**Figure 1:**
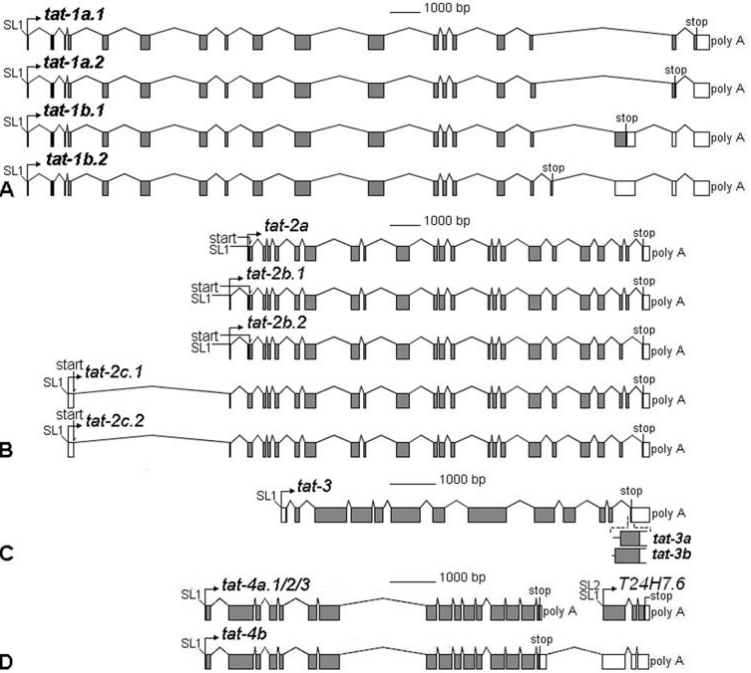
**Detected transcripts of *tat-1 *through *4***. While *tat-1 *(A) and *tat-2 *(B) consist of generally shorter exons and undergo significant alternative splicing, *tat-3 *(C) and *tat-4 *(D) include somewhat longer exons and encode essentially only one version of the product.

The *C. elegans *genome encodes six predicted members of the P-type ATPase subfamily IV. The genes are named *transbilayer amphipath transporter (tat*) *1 *through *6 *(Additional file 1). The four detected splice isoforms of *tat-1 *differ with respect to the final five exons (Figure 1A). Each isoform has a distinct stop codon and is predicted to generate a product with a divergent C terminus (Additional file 2). The five detected splice isoforms of *tat-2 *differ with respect to the first two and the penultimate exons and are predicted to generate four products with some sequence variability at the very N and C termini (Figure 1B). Only two slightly different isoforms of *tat-3 *were identified (Figure 1C). The product of the longer isoform contains a few extra C-terminal amino acids, which are absent in the shorter version. The *tat-4 *locus includes two open reading frames (ORFs), *tat-4 *and *T24H7*.*6*, which appear to form an operon (Figure 1D). *T24H7*.*6*, but not *tat-4*, cDNA could be amplified using a splice leader 2 (SL2) primer. In *C. elegan*s, *tran*s-splicing to SL2 usually indicates that a gene occupies a subordinate position in an operon [19]. The *tat-4 *stop codon resides in the same exon as at least three weak polyadenylation signals (Additional file 2). Bicistronic *tat-4 *and *T24H7.6 *messages were detected that terminate with the sole *T24H7.6 *polyadenilation signal and likely arise when the three *tat-4 *polyadenylation signals fail to induce poly(A) tail addition. The choice of polyadenylation site affects translation of neither *tat-4 *nor *T24H7*.*6*.

Three alternative transcripts of *tat-5 *were detected. *tat-5b *begins with the sequence from the two short exons located in a close proximity to the upstream ORF and undergoes *tran*s-splicing to SL2 (Figure 3A). *tat-5a *and *tat-5c *are almost identical to each other, start with the third exon located over 3 kilobases downstream from the first two *tat-5b *exons and are spliced to SL1 and SL2. In addition to *trans-*splicing to SL2, subordinate cistrons in an operon also usually reside close to the previous ORF [19]. By these two criteria, *tat-5b *is a subordinate cistron in an operon. The status of *tat-5a/c *is less certain. The long sequence from the end of the previous ORF to the start of *tat-5a/c *transcripts could conceivably hold another (in addition to the operon promoter) *cis-*acting regulatory element that drives transcription of these two isoforms.

**Figure 2:**
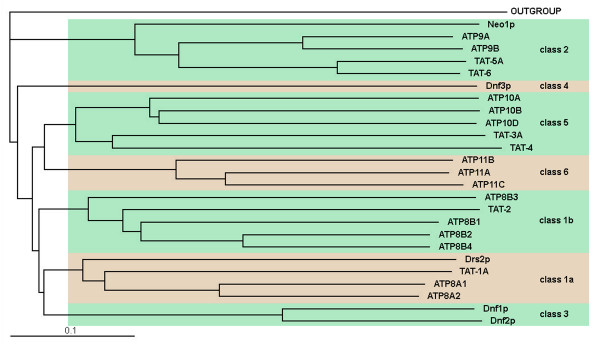
**A phylogenetic tree of *S. cerevisiae*, *C. elegans *and human P-type ATPases in subfamily IV.** The tree was assembled using ClustalW2 [48]. The outgroup is a *Drosophila melanogaster *calcium-transporting P-type ATPase.

**Figure 3:**
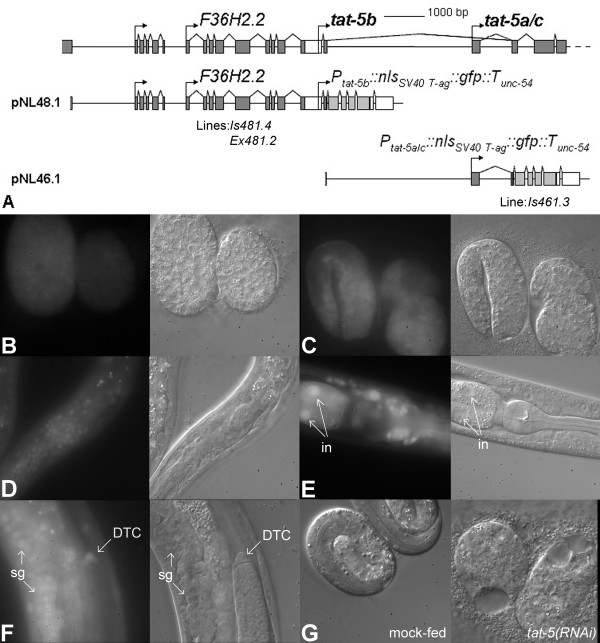
***tat-5 *is a housekeeping gene**. The 5' end of the *tat-5 *locus and structure of the two *tat-5 *expression cassettes (A). Expression of *tat-5b::nls::gfp *in the *lcIs481.4 *line in embryos (B and C) and a head region of an adult (D), and in the *lcEx481.2 *isolate at the head-intestine junction (E) and in a developing somatic gonad (F). Necrotic death of *tat-5(RNAi) *embryos (G). Abbreviations: DTC – distal tip cell; in – intestinal nucleus; sg – somatic gonad.

*tat-6 *is 73% identical with *tat-*5. A *tat-6 *deletion mutant (*ok1984*, a large middle portion of the protein product removed; see the *C. elegans *Gene Knockout Consortium) is reportedly viable. Comparatively weak expression of *tat-*6, evidenced by the paucity of the publicly available cDNA clones [20] and low cDNA amplification yield (data not shown), suggests that this gene is a recent poorly-expressed duplicate of the very strongly expressed *tat-*5. For these reasons, *tat-6 *was not characterized in detail.

P-type ATPases in subfamily IV are customarily divided into six classes [13]. A phylogenetic analysis of these ATPases expressed in *S. cerevisiae*, *C. elegans *and humans reveals a substantial evolutionary dissimilarity between class 2 and the other classes (Figure 2). After the early split between the branch leading to class 2 and the branch leading to the rest of the classes, class 2 genes have not duplicated significantly. Thus, yeast express a single gene in this class *(NEO1*), while *C. elegans *and humans express two class 2 genes each *(tat-5 *and *tat-6 *in the nematode). The other branch of the subfamily, in contrast, has quickly undergone multiplication and diversification. This difference in the extent of evolutionary expansion may indicate that the strictly preserved class 2 proteins perform some essential function conserved throughout evolution in all eukaryotes, while the frequently duplicating ATPases in the other classes rapidly evolve to fill new roles.

### *tat-5 *is a housekeeping gene

Two *tat-5 *expression cassettes were constructed (Figure 3A). In the *tat-5b::nls::gfp *cassette, a region of the operon promoter drives transcription of a sequence encoding a nuclear localization signal (NLS)-tagged green fluorescent protein (NLS-GFP). In *tat-5a/c::nls::gfp*, NLS-GFP is under the control of a fragment spanning the second *tat-5 *intron. The cassettes were introduced into the nematode genome via particle bombardment. Two transgenic lines – one integrated *(lcIs481.4*) and one extrachromosomal *(lcEx481.2*) – carrying *tat-5b::nls::gfp *transgenes were isolated (Figure 3A). In both lines GFP fluorescence emanates broadly from all inspected tissues at all developmental stages, except very early embryos and the germline (Figure 3B–F). One integrated line *(lcIs461.3*) carrying *tat-5a/c::nls::gfp *transgenes was identified. GFP signal could not be detected in *lcIs461.3 *embryos or hermaphrodites. These findings suggest that the operon promoter alone controls *tat-5 *transcription. The ubiquitous pattern of *tat-5 *expression revealed using the reporter is fully supported by *in situ *staining data from the nematode expression pattern database (NEXTDB [20]). Thus, *tat-5 *is a ubiquitously expressed gene.

N2 (wild-type) animals fed *tat-5 *double-stranded RNA (dsRNA) in order to suppress TAT-5 via RNA interference (RNAi) bore dead embryos showing signs of extensive necrosis (Figure 3G). Some eggs still in the uterus of *tat-5(RNAi) *hermaphrodites also seemed to disintegrate (data not shown). Similar *tat-5(RNAi) *phenotypes (hermaphrodite sterility and embryonic lethality) have been detected in systematic RNAi screens for genes whose suppression causes clear morphological and developmental abnormalities [21,22]. The three deletion mutants of *tat-5 (tm182*3, *tm1772 *and *tm1741*) are also reportedly homozygous lethal (see National Bioresource Project for the Nematode, Tokyo, Japan). Being ubiquitously expressed and essential for survival, *tat-5 *has the characteristics of a housekeeping gene.

### *tat-2 *through *4 *exhibit developmentally-regulated tissue-specific expression patterns

**Figure 4:**
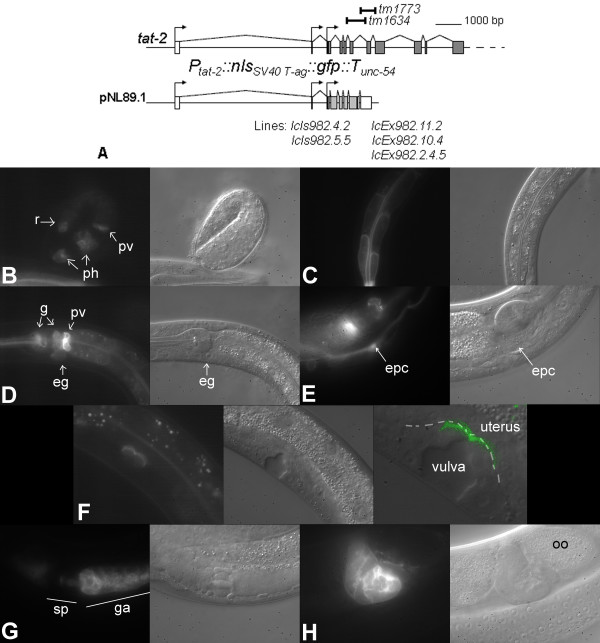
***tat-2 *reporter expression pattern**. The 5' end of the *tat-2 *locus, locations of the deleted regions in *tm1634 *and *tm1773 *mutants (bars) and structure of the *tat-2 *expression cassette (A). Expression of the *tat-2 *reporter in the *lcIs982.4.2 *line in embryos (B), in the intestine of L2 larvae (C) and in gland cells of the pharynx, the pharyngeal-intestinal valve, the excretory gland cell and the intestine of a young adult (D). GFP fluorescence in the *lcEx982.2.4.5 *line in the excretory pore and excretory canal cells (E). Reporter signal outlining the vulF cells in early *lcIs982.4.2 *L4 larvae (the third frame in the series shows an overlay of the pseudocolored UV image onto the bright light image, both enlarged with re-sampling using Photoshop; dashes extend along the contact surface between the two tissues) (F). *tat-2 *reporter expression during spermatogenesis (G) and in adult spermatheca (H) of *lcIs982.4.2 *animals. Abbreviations: eg – excretory gland cell; epc – excretory pore cell; g – pharyngeal gland cell; ga – gonad arm; ph – pharynx; pv – pharyngeal-intestinal valve; oo – oocyte; r – rectum; sp – spermatheca.

Two integrated and three extrachromosomal transgenic lines carrying *tat-2::nls::gfp *expression cassettes were made by particle bombardment (Figure 4A). With the exception of a few instances of ectopic transcription in the extrachromosomal lines, all five generally exhibit nearly identical patterns of reporter expression. Curiously, GFP fluorescence in the *tat-2::nls::gfp *transgenic nematodes emanates not from the nucleus, as would be expected with an NLS-tagged reporter, but mostly from the plasma membrane region (Figure 4B–H). Apparently, the short TAT-2-coding fragment that is retained in the *tat-2 *expression cassette "overpowers" the NLS and directs the chimeric reporter peptide to the plasma membrane compartment.

*tat-2 *reporter is first clearly detectable in 2-fold stage embryos in two sets of pharyngeal cells, the developing pharyngeal-intestinal valve and a set of cells in the posterior (Figure 4B). By the first larval (L1) stage, GFP fluorescence also appears in the intestine (Figure 4C). L4 and adult animals exhibit reporter signals in unidentified cells of the pharyngeal procorpus, the gland cells located in the posterior bulb of the pharynx, the pharyngeal-intestinal valve, rectal gland cells, the intestine and all cells of the excretory system (Figure 4D and 4E, and data not shown). *tat-2 *reporter signals are also seen in L4 larvae in the primary vulval lineage vulE and vulF cells and in the proximal gonad (Figure 4F and 4G). The vulval fluorescence vanishes and a moderately strong uterine signal appears after the uterine-vulval connection is complete in adults (data not shown). The gonadal signal, emanating from spermatids, migrates to the spermatheca around the time of the first ovulation (Figure 4G and 4H).

**Figure 5:**
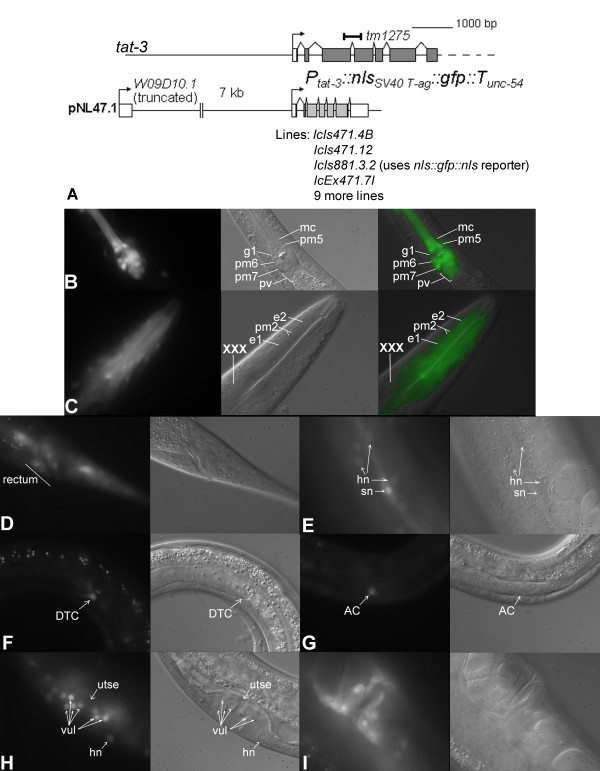
***tat-3 *reporter expression pattern**. The 5' end of the *tat-3 *locus, location of the deleted region in *tm1275 *mutants (bar) and structure of the *tat-3 *expression cassette (A). *tat-3 *reporter expression in the pharyngeal-intestinal valve and in muscle and marginal, but not gland, cells of the pharynx in the *lcIs471.12 *line (B). GFP fluorescence in the muscle and buccal epithelial cells of the pharynx procorpus and in the XXX cells in the *lcIs471.4B *line (C). *tat-3 *reporter signals in the rectum and a tail region (D), seam cells and the hypodermis (E), the DTC (F) and the AC (G) of *lcIs471.4B *animals. GFP staining of the vulva at the late L4 stage in *lcIs881.3.2 *larvae (H). Reporter expression in the adult *lcIs471.4B *vulva (I). Cell labels: AC – anchor cell; DTC – distal tip cell; e1 and 2 – buccal epithelial cells; g1 – gland cell; mc – marginal cell; pm2, 5, 6 and 7 – pharyngeal muscle cells; vul – vulval cells; utse – uterine seam cell; XXX – XXX cells. Abbreviations: hn – hypodermal nucleus; pv – pharyngeal-intestinal valve; sn – seam cell nucleus.

Three integrated and ten extrachromosomal transgenic lines carrying *tat-3::nls::gfp *cassettes were derived (Figure 5A). Four of these (all of the integrated and one extrachromosomal) were investigated in detail and found to exhibit nearly identical expression patterns. *tat-3 *reporter signal first appears in embryos in the developing pharynx (data not shown). In the fully formed alimentary system, very strong GFP fluorescence is observed in the muscle, marginal and buccal epithelial cells of the pharynx, the pharyngeal-intestinal valve and, with lesser intensity, the rectal epithelial cells (Figure 5B–D). Seam cells display very strong fluorescence as soon as this lineage becomes established during embryonic development (Figure 5E). In adults, moderate to weak fluorescence seems to arise from the XXX cells, some unidentified cells in the head and tail regions and the hypodermis (Figure 5C and 5E). In the reproductive system, *tat-3 *reporter expression begins in the distal tip cells (DTC) in L1 and in the anchor cell (AC) in early L3 (Figure 5F and 5G). GFP signal is later visible in the dividing progeny of the vulval precursor cells (VPCs). In late L4, the anchor cell fuses with the uterine seam cell (utse), which does not express the reporter (Figure 5H). The vulval cells continue exhibiting moderate fluorescence into the adulthood (Figure 5H and 5I).

**Figure 6:**
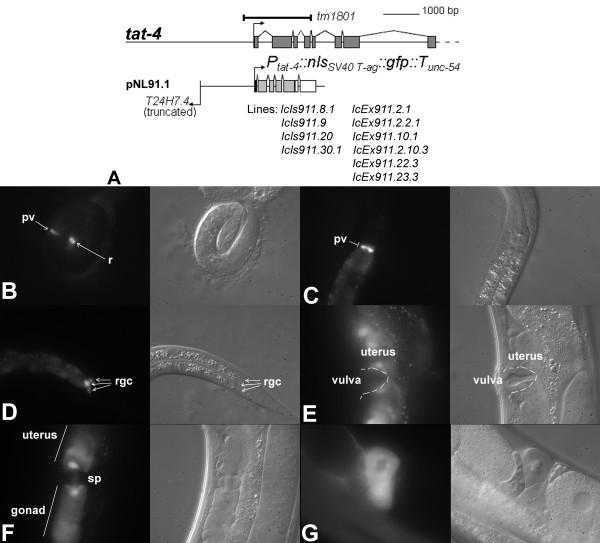
***tat-4 *reporter expression pattern**. The 5' end of the *tat-4 *locus, location of the deleted region in *tm1801 *mutants (bar) and structure of the *tat-4 *expression cassette (A). Reporter expression in the pharyngeal-intestinal valve, intestine and rectal gland cells in 3-fold embryos (B) and in advanced stage larvae (C and D) in the *lcIs911.30 *line. GFP fluorescence in the uterus (E), during spermatogenesis (F) and in the spermatheca of previously ovulated hermaphrodites (G) in *lcIs911.30 *animals. Abbreviations: pv – pharyngeal-intestinal valve; r – rectum; rgc – rectal gland cells; sp – spermatheca.

Four integrated and six extrachromosomal *tat-4::nls::gfp *transgenic lines were derived (Figure 6A). Five of these lines (2 integrated and 3 extrachromosomal) were investigated in detail. Notable *tat-4 *reporter expression begins in 2–3 fold embryos in the developing pharyngeal-intestinal valve and unidentified cells at the posterior (Figure 6B). In the fully formed alimentary system, GFP fluorescence emanates strongly from the pharyngeal-intestinal valve, rectal gland cells and the intestine (Figure 6C and 6D). *tat-4 *reporter is also expressed in the uterus, during spermatogenesis in the proximal gonad and in the spermatheca of previously ovulated adults (Figure 6E–6G).

The available from NEXTDB [20]* in situ *staining images corroborate expression of *tat-3 *in the pharynx and vulva, of *tat-4 *in the spermatheca, intestine, pharyngeal-intestinal valve and uterus and of *tat-2 *in the intestine. *tat-1 *expression pattern could not be obtained because three near perfect inverted repeats located in the 5' end of the *tat-1 *locus destabilized expression cassette vectors (data not shown). However, images from NEXTDB show that *tat-1 *is also expressed tissue-specifically. Thus, *tat-2*, *tat-3*, *tat-4 *and, likely, *tat-1 *all appear to be expressed in developmentally regulated tissues-specific patterns (Table 1).

**Table 1:** Expression of *tat-2*, *tat-3*, *tat-4 *and *tat-5 *in *C. elegans *tissues.

Cells and tissues	*tat-2*	*tat-3*	*tat-4*	*tat-5*
All somatic tissues				+++
Germline	-	-	-	-
				
Alimentary system				
Pharyngeal gland cells	+++	-	-	
Pharyngeal muscle cells	-	+++	-	
Pharyngeal marginal cells	-	+++	-	
Buccal epithelium	-	+++	-	
Pharyngeal-intestinal valve	+++	+++	+++	
Intestine	+++	-	+++	
Rectal gland cells	+++	-	+++	
Rectal epithelial cells	-	++	-	
				
Reproductive system				
DTCs	-	+++	-	
AC	-	+++	-	
Developing vulva: vulE and vulF cells only	+++	+++	-	
Developing vulva: VPC progeny	-	+++	-	
Adult vulva	-	++	-	
Uterus	++	-	+++	
Spermatogenesis	+++	-	+++	
Adult spermatheca	+++	-	+++	
				
Excretory system	+++	-	-	
				
Epithelial system				
Seam cells	-	+++	-	
Hypodermis	-	++	-	
				
Other				
Head and tail region cells/XXX cells	-	++	-	

Expression pattern of *tat-1 *could not be determined by our method of choice, and *tat-6 *was excluded because this gene appears to be a recent poorly expressed duplicate of *tat-5*.

### *tat-1 *through *4 *are nonessential under regular growth conditions

TAT-2 through 4 expression patterns show that these proteins are present in critical tissues during key periods of the nematode development. To determine whether curtailing expression of *tat-1*, *tat-2*, *tat-3 *or *tat-4 *would lead to gross morphological and developmental abnormalities, N2 animals were fed dsRNA against the four genes. *tat-1(RNAi*), *tat-2(RNAi*), *tat-3(RNAi) *and *tat-4(RNAi) *animals did not exhibit a notable developmental or morphological defect (data not shown). However, staining of germ line apoptotic cells with annexin V-GFP, a peptide that binds specifically PS, was altered in *tat-1(RNAi) *hermaphrodites [23], suggesting that RNAi against *tat-1 *did suppress its target.

While the RNAi studies were being conducted, deletion mutants of *tat-2 *through *4 *became available. *tat-2(tm1773) *(frame shift, splicing acceptor deleted), *tat-3(tm1275) *(frame shift) and *tat-4(tm1801) *(a basal promoter region and a large portion of the coding sequence deleted) are very likely null (Figure 4A, Figure 5A and Figure 6A). *tat-2(tm1634) *lacks an N-terminal exon encoding 44 amino acids present in all isoforms, but may still be partly functional (Figure 4A). *tat-2(tm1773*), *tat-2(tm1634*), *tat-3(tm1275) *and *tat-4(tm1801) *single and *tat-4(tm1801); tat-3(tm1275) *double mutants are all viable.

**Figure 7:**
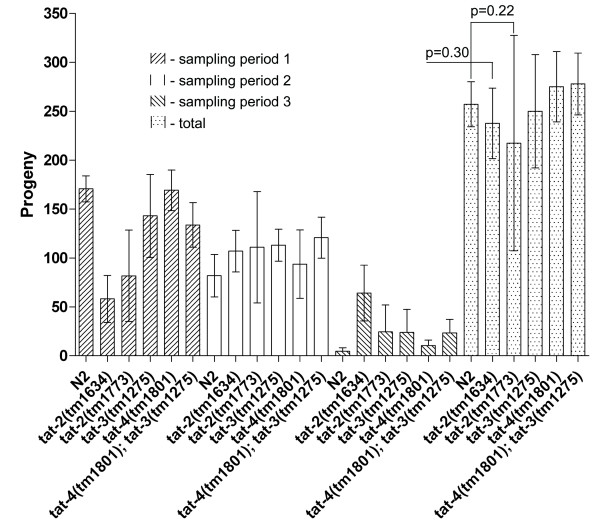
**Reproduction of mutant and N2 nematodes under regular growth conditions** (n = 10 to 12 animals; statistical analysis performed using ANOVA; error bars are standard deviations).

To determine whether deletion of *tat-2 *through *4 *exerts a negative effect on nematode growth and reproduction, synchronized mutant and N2 larvae were followed through developmental stages, and the viable progeny of the adult hermaphrodites were counted. Wild-type, *tat-3(tm1275*), *tat-4(tm1801) *and *tat-4(tm1801); tat-3(tm1275) *animals were essentially indistinguishable from one another in both the timing of progression through the developmental stages and the number of viable progeny produced by hermaphrodites (Figure 7). *tat-2(tm1634) *mutants passed through development slightly slower than un-mutated larvae. This is evident in the lower and higher number of *tat-2(tm1634) *offspring produced during, respectively, the first and the last sampling period, in comparison with the numbers of N2 progeny. Around 20% (n = 10) of *tat-2(tm1773) *hermaphrodites had notably fewer progeny than the rest of animals of the same genotype. This is reflected in the large standard deviation value for this mutant. However, pair-wise statistical analysis (ANOVA) shows that the total number of viable hatchlings for neither *tat-2(tm1634) *nor *tat-2(tm1773) *mutants was significantly different from the corresponding number for N2 animals (P = 0.22 and P = 0.30, respectively). Overall, deletion of *tat-1*, *tat-2*, *tat-3 *or *tat-4 *individually and *tat-3 *and *tat-4 *together does not seem to impair nematode development or reproduction to a significant degree under regular growth conditions.

### *tat-2 *and *tat-4 *mutant animals are hypersensitive to sterol deprivation

*C. elegans *is a sterol heterotroph that uptakes various exogenous sterols and converts these compounds to 7-dehydrocholesterol [24-26]. The latter metabolite is a precursor of dafachronic acids, a hormone that promotes reproductive growth [27]. Sterol uptake and conversion to 7-dehydrocholesterol occurs in the intestine [28-30], while dafachronic acids are synthesized primarily in the hypodermis [31]. If subfamily IV P-type ATPases TAT-2 through 4 facilitated a step somewhere along the sterol transport pathway – from the site of exogenous sterol uptake to the site of 7-dehydrocholesterol conversion to dafachronic acids, then *tat-2 *through *4 *mutants would exhibit sterol deprivation hypersensitivity evident in decreased reproductive growth.

The solid support medium for routine nematode growth contains sterols from the substances used in its preparation and is also supplemented with cholesterol to the final concentration of 5000 ng/ml [32]. The combined amount of sterol in the medium is more than sufficient for optimal nematode growth: N2 animals can grow just as well on plates enriched with cholesterol to 1000 ng/ml [30]. To determine whether *tat-2 *through *4 *mutants are hypersensitive to cholesterol deprivation, test plates were specially prepared to eliminate all exogenous sources of sterol and then supplemented with cholesterol to the final concentrations of 1000 ng/ml, 100 ng/ml, 10 ng/ml or 1 ng/ml. OP50 strain *Escherichia coli *cultures were grown in a synthetic defined medium without sterol, then supplemented with cholesterol to the same concentrations as the plates. Test plates were spotted with a bacterial culture of the same cholesterol concentration. The resultant food lawns on the test plates were almost identical in size. Eggs from gravid hermaphrodites grown on regular full-sterol plates were collected using the alkaline hypochlorite method, hatched overnight on no-sterol plates, and then the hatchlings were transferred to test plates, 30 per plate (on day one). The first generation larvae on the test plates grew to maturity and reproduced because these animals contained reserve sterol deposited into oocytes by the mothers maintained on the regular high supply of the nutrient [29]. The second generation lacked sterol reserves and exhibited notable effects of sterol deprivation.

**Figure 8:**
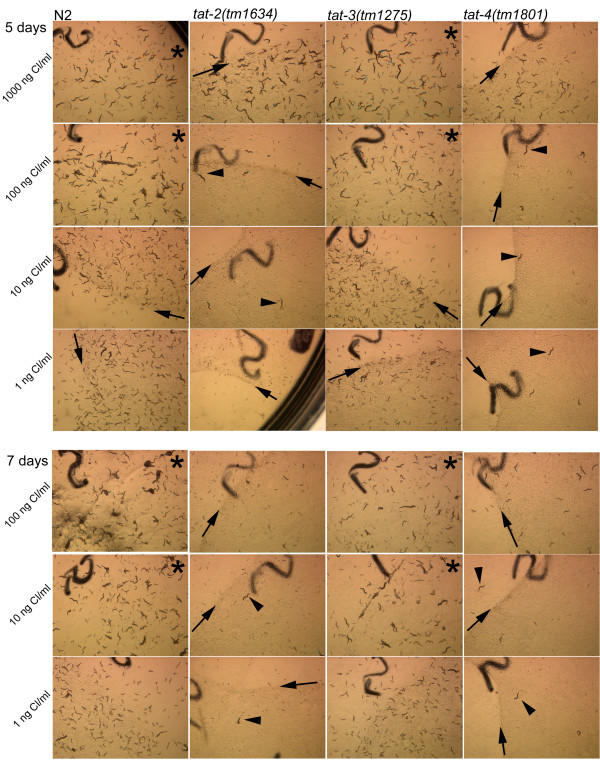
**Sterol deprivation sensitivity of mutant and N2 nematodes**. Notations: arrows indicate the edge of food lawn on plates that still have bacterial food; arrow heads point to the first generation animals; stars identify plates that began starving. The dark lines on the photographs are numbers written on the underside of plates.

Growth of N2 animals on the test plates was proportional to the amount of cholesterol in the medium (Figure 8). By the fifth day, N2 populations on 1000 ng/ml and 100 ng/ml cholesterol plates cleared all bacterial food and began starving. This indicates that the first generation animals produced plenty of viable progeny and that the second generation grew quickly without significant mortality. N2 populations on 10 ng/ml cholesterol plates cleared food on day 7. By this same time, N2 1 ng/ml cholesterol plates still contained plenty of food and fewer and much smaller second-generation animals.

*tat-3(tm1275) *populations grew on the test plates at the same pace as the wild-type populations. In contrast, *tat-2(tm1634) *and *tat-4(tm1801) *populations exhibited a much more dramatic retardation of growth (Figure 8). Food on *tat-2(tm1634) *and *tat-4(tm1801) *1000 ng/ml cholesterol plates was cleared on day 7: two days later than on the same cholesterol concentration N2 and *tat-3(tm1275) *plates. Significantly, on 100 ng/ml and lower cholesterol concentration plates, growth of the second generation *tat-2(tm1634) *and *tat-4(tm1801) *larvae progressed minimally. This is evident in slight, if any, changes in the size of *tat-2(tm1634) *and *tat-4(tm1801) *populations and individual animals from day 5 to 7 (Figure 8). *tat-2(tm1634) *and *tat-4(tm1801) *mutants did not appear to suffer from a particular developmental or morphological defect but rather respond to sterol limitation more dramatically than N2 and *tat-3 *animals. Furthermore, the developmental delay exhibited by *tat-2(tm1634) *animals on the sterol-limited test plates was much more severe than the mild lag in development these mutants showed on regular plates. *tat-2(tm1773) *populations also exhibited severe growth retardation on sterol-limited test plates (data not shown). Since it is unlikely that three independently derived deletion alleles in two related genes would contain secondary mutations that cause an identical phenotype, mutagenesis of specifically *tat-2 *and *tat-4 *seems to induce hypersensitivity to sterol deprivation.

### *tat-4(tm1801); tat-3(tm1275) *double mutants are more sensitive to sterol availability than *tat-4(tm1801) *single mutants

**Figure 9:**
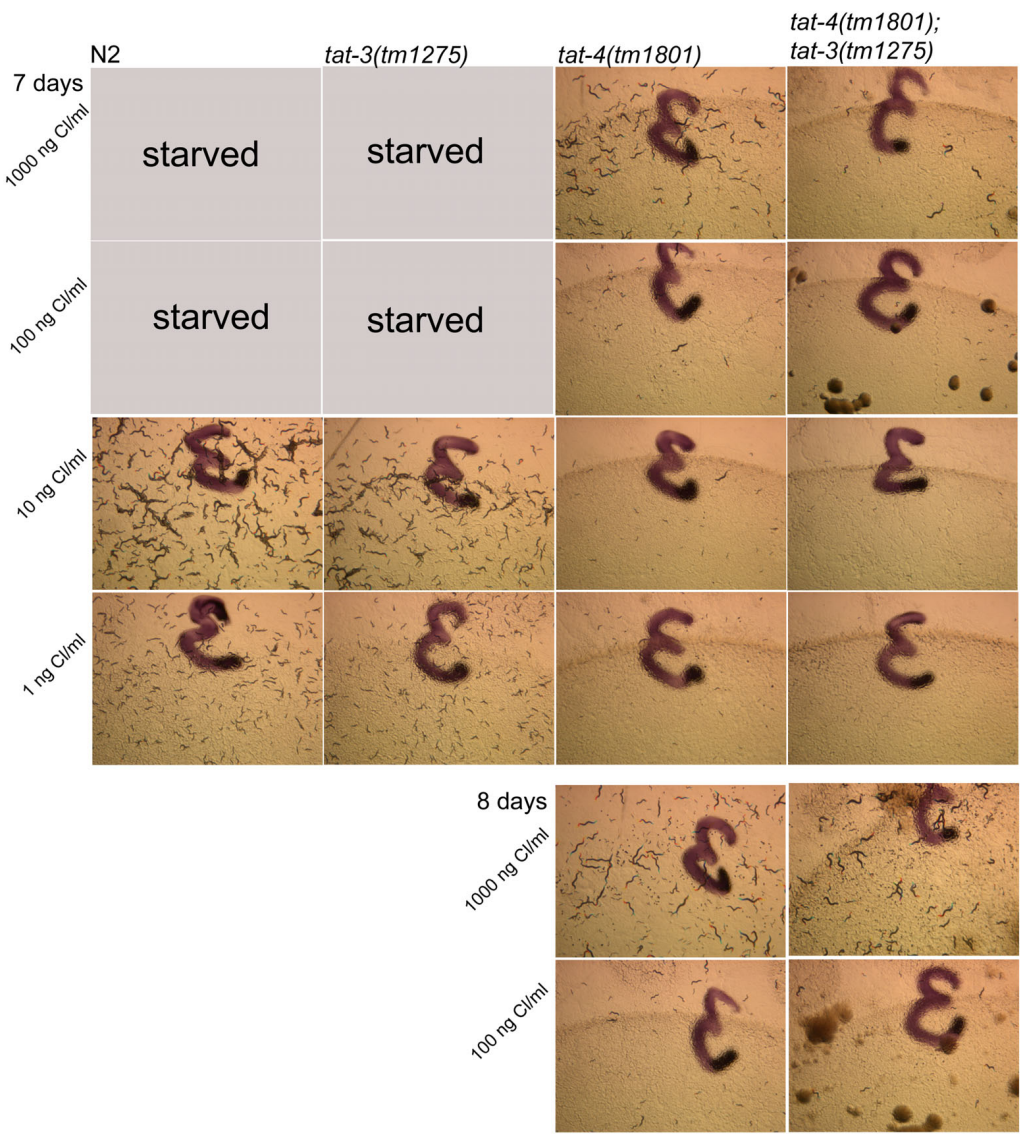
**Higher sensitivity to sterol limitation of *tat-4(tm1801); tat-3(tm1275) *double mutants in comparison with *tat-4(tm1801) *single mutants**. The dark lines on the photographs are numbers written on the underside of plates.

The above data suggest that *tat-3 *is not a major player in sterol metabolism; however, the gene is expressed in the hypodermis, a major steroidogenic tissue [28], and in the pharynx, a potential sterol storage organ [32]. To test whether *tat-3 *contributes marginally to sterol metabolism, *tat-3(tm1275) *mutants were crossed with *tat-4(tm1801) *mutants to derive a double mutant of the two genes. *tat-3 *and *tat-4 *expression patterns overlap only in the pharyngeal-intestinal valve (Table 1), and in terms of development and reproduction under regular conditions, *tat-4(tm1801); tat-3(tm1275) *animals are indistinguishable from N2 nematodes (Figure 7). On sterol-deprivation test plates, double mutants of the two genes performed worse than *tat-4(tm1801) *nematodes (Figure 9). *tat-4(tm1801) *populations were noticeably larger than *tat-4(tm1801); tat-3(tm1275) *populations by day 7 on 1000 ng/ml cholesterol concentration plates. By day 8 on the same plates, *tat-4(tm1801) *animals cleared all food, while there were still plenty of bacteria to consume for the double mutants. Growth was minimal on 100 ng/ml and lower cholesterol concentration plates for both *tat-4(tm1801); tat-3(tm1275) *and *tat-4(tm1801) *animals during the 9-day observation period. Thus, it seems deletion of *tat-3 *exacerbates sterol-deprivation hypersensitivity of *tat-4 *mutants.

## Discussion

Specialization and redundancy among the *C. elegans *putative aminophospholipid translocases

Eukaryotic genomes contain many genes encoding subfamily IV P-type ATPases [13]. The need for such an abundance of these genes per organism and their individual functions are presently poorly understood. Closely related genes may be at least partly redundant or they may be functionally highly specialized. The *C. elegans *genome includes six, *tat-1 *through *6*, genes whose protein products are P-type ATPases in subfamily IV. The data presented here indicate that *tat-5 *encodes the sole ubiquitously expressed and essential subfamily IV P-type ATPase. *tat-5(RNAi) *phenotype is dramatic: sterility and embryonic lethality; furthermore, tissues of *tat-5(RNAi) *embryos exhibit extensive necrotic cell death. These observations suggest that TAT-5 performs a very critical housekeeping function. Together with the yeast Neo1p [14], which is also essential, TAT-5 segregates in class 2, the most ancient class of P-type ATPases in subfamily IV. Thus, it seems that class 2 genes encode highly specialized subfamily IV P-type ATPases that execute a critically important housekeeping function.

Individual deletion of the remaining *C. elegans *subfamily IV P-type ATPase genes, *tat-1 *through *4*, (excluding *tat-6*, a recent poorly expressed duplication of *tat-5*) does not grossly impair *C. elegans *growth and development. This would be consistent with a high degree of redundancy among these genes. However, an analysis of expression and involvement in sterol metabolism exhibited by *tat-2 *through *4 *suggests a different explanation. While *tat-4 *is expressed in the same tissues as *tat-2*, *tat-3 *expression occurs in almost completely different tissues than those expressing either *tat-2 *and *tat-4 *or *tat-2 *alone (Table 1). Furthermore, *tat-4; tat-3 *double mutants are just as viable as *tat-4 *and *tat-3 *single mutants and wild-type animals. These observations indicate that although closely related (both in class 5), *tat-3 *and *tat-4 *are not redundant. Although the less closely related *tat-2 *(class 1b) and *tat-4 *are expressed in the same tissues, deletion of either one confers hypersensitivity to sterol deprivation to the same extent, which suggests that the two at most only partly redundant. *tat-2 *is the only gene of the three expressed in the excretory system. Curiously, while *tat-3 *expression occurs in all VPC progeny during vulval development and then continues on in the adult vulva, *tat-2 *expression takes place specifically in the vulE and vulF cells during vulval development only. This suggests that the two genes perform different roles in the development of the vulva. Expression pattern of *tat-1 *(class 1a) could not be obtained with our method of choice (transcriptional reporters stably introduced into the genome using particle bombardment). However, the data presented by Darland-Ransom et al. [6] indicate that this gene is expressed in the gonad and body-wall muscle cells – tissues in which *tat-2 *through *4 *are not expressed. Overall, the lack of gross growth and development impairment in *tat-1 *through *4 *mutants does not seem to stem from a high level of redundancy among these genes.

While the individually dispensable *S. cerevisiae *subfamily IV P-type ATPase genes (*DRS2*, *DNF1*, *DNF2 *and *DNF3*) are essential as a group [17], the individually dispensable *C. elegans *subfamily IV P-type ATPase genes may also be dispensable as a group. *tat-1 *seems to be the only dispensable subfamily IV P-type ATPase expressed in the body-wall muscle cells [6]. *tat-1 *null animals lose a few extra muscle cells, but this does not translate into a notable growth defect [6]. This suggests that together the four class 1 and 5 subfamily IV P-type ATPases are nonessential for routine growth and development of *C. elegans *cells.

Investigations in *S. cerevisiae *suggest that individually dispensable subfamily IV P-type ATPases are redundant, especially with respect to internalization of PS and PE [17]. However, this does not seem to be the case for individually dispensable *C. elegans *subfamily IV P-type ATPases. Considering that *C. elegans *germline and somatic cells normally conceal PS [23] and that loss of *tat-1 *leads to PS appearance on the surface of germline and certain somatic cells [6], the contradiction between the yeast and the nematode paradigms does not imply that subfamily IV P-type ATPases of the latter organism do not translocate aminophospholipids. Rather, considering that class 2 subfamily IV members may execute the same biochemical functions as subfamily IV members in other classes [14] and that loss of asymmetric PS and PE distribution across the plasma membrane is incompatible with survival [33], our findings suggest that, in the nematode, TAT-5 carries out the bulk of aminophospholipids internalization, while TAT-1 through 4 adjust cross-bilayer lipid distribution in particular tissues.

### *C. elegans *subfamily IV P-type ATPases fine-tune sterol metabolism

*C. elegans *is a sterol heterotroph that employs a steroid-mediated developmental program. The nematode takes up exogenous sterols in the intestine and then converts them to 7-dehydrocholesterol [28]. The latter metabolite is used for synthesis of the reproductive development-promoting hormone, dafachronic acids, in the hypodermis [27,28]. In unfavorable growth conditions, production of the hormone stops and nematode larvae embark on the dauer (dispersal) developmental pathway. Complete sterol deprivation mimics cessation of the hormone synthesis in an otherwise favorable growth environment [34]. At sterol supplies below optimal, nematode development and reproduction are proportional to sterol concentration in the medium [30]. As a result, *C. elegans *reproductive growth is a sensitive indicator of the sterol level in the hypodermis after this lipid was taken up in the intestine and then traveled through the animal body.

Our data show that *tat-2 *and *tat-4 *are required for growth during sterol starvation and that deletion of *tat-3 *exacerbates sterol hypersensitivity of *tat-4 *mutants. From the late stages of embryonic development through the rest of the nematode life cycle, *tat-2 *and *tat-4 *are strongly expressed in the intestine and other cells of the alimentary system; it is likely that the protein products of these genes fine-tune some aspects of sterol processing in these sterol-metabolizing tissues. *tat-2 *is a homolog of the mammalian *Atp8b1/ATP8B1*, which encodes subfamily IV P-type ATPase that localizes to the apical membrane of hepatocytes, enterocytes and other polarized cells of the mammalian digestive system [35,36]. Future investigations should determine whether TAT-2 also localizes to the apical membrane and whether TAT-4 resides in the same membrane as TAT-2. *tat-3 *is expressed in the hypodermis and the XXX cells, both steroidogenic tissues where 7-dehydrocholesterol is converted to dafachromic acids [28]. Thus, it is likely that the product of this gene mediates, at least partly, turnover of 7-dehydrocholesterol after the release of this metabolite from the intestine. Future research should test this hypothesis, as well as determine the membrane compartment in which TAT-3 resides. Overall, the lack of a notable phenotype in *tat-1(RNAi) *and *tat-2 *through *4 *null mutants during growth under normal conditions and the inability of *tat-2 *and *tat-4 *mutants to grow under sterol limiting conditions indicate that one critical function of the dispensable subfamily IV P-type ATPases may be to fine tune metabolism of sterol and, perhaps, other lipids.

Recent reports indicate that the yeast *DRS2 *is also involved in sterol (ergosterol) metabolism [37-39]. *DRS2Δ *mutants exhibit reduced ability to uptake exogenous sterol. *Atp8b1/ATP8B1 *mediates sterol (cholesterol) metabolism in mammals [40,41]. Mutants of *Atp8b1 *secrete more cholesterol with bile [40]. And homozygous individuals with compromised *ATP8B1 *exhibit reduced levels of high-density lipoprotein (HDL)-cholesterol and greatly increased levels of oxidized cholesterol [41]. Together with these previous findings in yeast and mammals, involvement of *tat-2 *through *4 *in sterol metabolism in *C. elegans *suggests that the role performed by the subfamily IV P-type ATPases in this process may be conserved among eukaryotes and far greater than currently recognized.

## Conclusion

The presented study shows that individually dispensable *C. elegans *putative aminophospholipid translocase genes exhibit a high degree of specialization. This finding contradicts the expectation derived from investigations in yeast that these genes should be broadly redundant and answers the question of why there are so many genes of this kind per organism. Our study also partly answers the question of individual roles performed by putative aminophospholipid translocases by uncovering widespread involvement of members of this group in sterol metabolism.

## Methods

### *C. elegans *culture and genetics

The following strains and mutants of *C. elegans *were used: var. Bristol strain N2 (wild type), *unc-119(-), tat-2(tm1634) IV, tat-2(1773) IV, tat-3(tm1275) III, tat-4(tm1801) II *and *tat-4(tm1801) II; tat-3(tm1275) III*. The 4 *tat *mutants were isolated in the var. Bristol strain N2 background by the National Bioresource Project for the Experimental Animal Nematode [42]. The mutants were out-crossed 2 to 8 times to N2 animals. Except where noted, *C. elegans *animals were maintained on standard nematode growth medium plates spotted with *E. coli *OP50 at 20°C [32].

### Gene transcription analysis

Mixed-stage N2 animals were grown on enriched peptone plates. Populations approaching starvation were collected, combined, re-suspended in Trizol^® ^(Invitrogen, Carlsbad, CA), freeze-thawed three times and treated to 20 strokes of the A pestle in a 40 ml Dounce homogenizer. Total RNA was then isolated as recommended by the Trizol manufacturer. mRNA was purified from the total RNA with a Poly(A)Pure kit (Ambion, Austin, TX). cDNA was synthesized using either the dT(18) or the adaptor-dT(18) primers (see Additional file 4). For 5' RACE, a splice leader-specific primer and a gene-specific primer (GSP) were employed; for 3' RACE, a GSP and the adaptor-dT(18) primer were used. Products of cDNA amplification were resolved on aragose gels, purified and cloned into a TA helper vector. A number of clones for each distinct band on the agarose gels were sequenced (see Additional file 4). The following ESTs kindly provided by Dr. Kohara were sequenced: yk1228h06, yk34c11, yk1496c06, yk209d5, yk36b8, yk214f10 and yk126b10.

### Gene expression analysis

Nucleus-targeted transcriptional reporters were used to visualize *tat-2 *through *5 *expression. This approach was chosen because *tats *are very long genes; a tag often destabilizes P-type ATPases in subfamily IV ([14] and Lyssenko, unpublished observations); in yeast and mammals, members of the subfamily exit the Golgi apparatus only as a heterodimer with proteins in the Cdc50p/Lam3p group [7,12], over-expression of the *tats *may lead to accumulation of un-paired peptide in the Golgi apparatus and potentially induction of the unfolded protein response, which could then trigger apoptosis of the reporter-expressing cells; and finally, concentrating reporters in the nucleus facilitates cell identification [43]. Note also that when long portions (long enough to code for over 65 amino acids) of *tat-2 *and *tat-4 *translated sequences were included into expression cassettes, these cassettes did not assemble into extrachromosomal arrays and did not integrate into the genome (Additional file 3).

Chromosomal fragments that contain promoter regions of *tat-2 *through *5 *were cloned from the YAC Y17G9 and cosmids T24H7, W09D10 and F36H2 into pPD95.69, a promoter-less NLS-GFP expression vector from the Fire vector kit, using standard cloning methods (see Additional file 4). DNA of an expression vector was co-precipitated with DNA of a vector carrying *unc-119(+) *(pDP#MM016B [44]) onto gold particles as described by Tucker et al. [45], and then the coated particles were bombarded into the gonad of *unc-119(-) *nematodes as described by Praitis et al. [46] with some modifications (see Additional file 4). After bombardment, nematodes were washed off the plate and transferred to 2 or 3 fresh plates. Following an incubation period, stably transgenic lines (not exhibiting the Unc phenotype) were selected from the plates: only one hermaphrodite from each plate with non-Unc animals. Integrated lines were identified by whether animals homozygous for the trangene could be derived.

### RNA interference

500 to 900 bp-long 5' fragments of *tat-1 *through *5 *cDNA clones were sub-cloned into pPD129.36 (inverted-T7-promoter vector from the Fire vector kit). dsRNA production and nematode feeding were conducted as described in Kamath et al. [47] (see Additional file 4).

### Growth and reproduction under regular conditions

Gravid N2 and mutant hermaphrodites were allowed to lay eggs on fresh plates for 2 h. The animals were then removed, and the eggs left to hatch overnight. The next day, 10–12 larvae per genotype were transferred to individual plates: one larva per plate. 94 and 118 h later, the now adult nematodes were transferred to new plates. The old plates were incubated overnight to permit all viable eggs to hatch, chilled on ice to halt nematode movement, and then the larvae were counted. 0–94 h, 94–118 h and 118–154 h are sampling periods one, two and three, respectively.

### Sterol deprivation assay

A mixture of agarose (8 g/l) and ether-extracted peptone in water was autoclaved for 30 min, cooled to 65°C in a water bath and enriched to the desired concentrations with 5 mg/ml cholesterol in ethanol. An OP50 strain *E. coli *culture was grown in a minimal medium (20 mM NH_4_Cl, 0.2% w/v D-glucose, 2 mg/ml uracil in M9 buffer) overnight at 37°C. The culture was divided into 4 aliquots, which were centrifuged to remove the supernatant, re-suspended in the same volume of peptone solution (0.5% w/v peptone in M9 buffer) and enriched with cholesterol to the same 4 final concentrations as the plates. The suspensions were then pipetted carefully onto the corresponding concentration plates to produce uniform spots. Nematodes were synchronized at L1 by hatching eggs on no-sterol plates. L1 larvae were then transferred to spotted plates, exactly 30 animals per plate. 3 replica plates were seeded per cholesterol concentration per genotype. The populations were allowed to grow at 20°C for 1.5–2 weeks with periodic observations.

## Authors' contributions

NNL conceived the project, designed experiments, performed experiments, analyzed data and wrote the manuscript. YN performed experiments and analyzed data. SG, WH and RAS contributed to experimental design, analyzed data and edited the manuscript. All authors read and approved the final manuscript.

## Supplementary Material

Additional file 1 WormBase IDs and given names of the six *C. elegans *ORFs encoding P-type ATPases in subfamily IV. Click here for file

Additional file 2 Peculiarities of *tat-1 *and *tat-4 *transcription. Click here for file

Additional file 3 Fusions of GFP with long N-terminal portions of either TAT-2 or TAT-4 were not expressed due to apparent toxicity. Click here for file

Additional file 4 Supplementary materials and methods. Click here for file

## References

[B1] Lenoir G, Williamson P, Holthuis JCM (2007). On the origin of lipid asymmetry: the flip side of ion transport. Curr Opin Chem Biol.

[B2] Tang X, Halleck MS, Schlegel RA, Williamson P (1996). A subfamily of P-type ATPases with aminophospholipid transporting activity. Science.

[B3] Axelsen KB, Palmgren MG (1998). Evolution of substrate specificities of the P-type ATPase superfamily. J Mol Evol.

[B4] Ding J, Wu Z, Crider BP, Ma Y, Li X, Slaughter C, Gong L, Xie XS (2000). Identification and functional expression of four isoforms of ATPase II, the putative aminophospholipid translocase. Effect of isoform variation on the ATPase activity and phospholipid specificity. J Biol Chem.

[B5] Paterson JK, Renkema K, Burden L, Halleck MS, Schlegel RA, Williamson P, Daleke DL (2006). Lipid specific activation of the murine P4-ATPase Atp8a1 (ATPase II). Biochemistry.

[B6] Darland-Ransom M, Wang X, Sun CL, Mapes J, Gengyo-Ando K, Mitani S, Xue D (2008). Role of *C. elegans *TAT-1 protein in maintaining plasma membrane phosphatidylserine asymmetry. Science.

[B7] Paulusma CC, Folmer DE, Ho-Mok KS, de Waart DR, Hilarius PM, Verhoeven AJ, Oude Elferink RP (2008). ATP8B1 requires an accessory protein for endoplasmic reticulum exit and plasma membrane lipid flippase activity. Hepatology.

[B8] Iwamoto K, Kobayashi S, Fukuda R, Umeda M, Kobayashi T, Ohta A (2004). Local exposure of phosphatidylethanolamine on the yeast plasma membrane is implicated in cell polarity. Genes Cells.

[B9] Wang L, Beserra C, Garbers DL (2004). A novel aminophospholipid transporter exclusively expressed in spermatozoa is required for membrane lipid asymmetry and normal fertilization. Dev Biol.

[B10] Pomorski T, Lombardi R, Riezman H, Devaux PF, van Meer G, Holthuis JC (2003). Drs2p-related P-type ATPases Dnf1p and Dnf2p are required for phospholipid translocation across the yeast plasma membrane and serve a role in endocytosis. Mol Biol Cell.

[B11] Kuhlbrandt W (2004). Biology, structure and mechanism of P-type ATPases. Nat Rev Mol Cell Biol.

[B12] Chen S, Wang J, Muthusamy BP, Liu K, Zare S, Andersen RJ, Graham TR (2006). Roles for the Drs2p-Cdc50p complex in protein transport and phosphatidylserine asymmetry of the yeast plasma membrane. Traffic.

[B13] Halleck MS, Schlegel RA, Williamson PL (2002). Reanalysis of *ATP11B*, a type IV P-type ATPase. J Biol Chem.

[B14] Wicky S, Schwarz H, Singer-Krüger B (2004). Molecular interactions of yeast Neo1p, an essential member of the Drs2 family of aminophospholipid translocases, and its role in membrane trafficking within the endomembrane system. Mol Cell Biol.

[B15] Hua Z, Fatheddin P, Graham TR (2002). An essential subfamily of Drs2p-related P-type ATPases is required for protein trafficking between the Golgi complex and endosomal/vacuolar system. Mol Biol Cell.

[B16] Natarajan P, Wang J, Hua Z, Graham TR (2004). Drs2p-coupled aminophospholipid translocase activity in yeast Golgi membranes and relationship to *in vivo *function. Proc Natl Acad Sci USA.

[B17] Graham TR (2004). Flippases and vesicle-mediated protein transport. Trends Cell Biol.

[B18] Schlegel RA, Williamson P (2001). Phosphatidylserine, a death knell. Cell Death Differ.

[B19] Blumenthal T (1995). *Trans*-splicing and polycistronic transcription in *Caenorhabditis elegans *operons. Trends Genet.

[B20] Shin-i T, Kohara Y (1999). NEXTDB: the expression pattern map database for *C. elegans*. Genome Informatics.

[B21] Fraser AG, Kamath RS, Zipperlen P, Martinez-Campos M, Sohrmann M, Ahringer J (2000). Functional genomic analysis of *C. elegans *chromosome I by systemic RNA interference. Nature.

[B22] Maeda I, Kohara Y, Tamamoto M, Sugimoto A (2001). Large-scale analysis of gene function in *Caenorhabditis elegans *by high-throughput RNAi. Curr Biol.

[B23] Züllig S, Neukomm LJ, Jovanovic M, Charette SJ, Lyssenko NN, Halleck MS, Reutelingsperger CP, Schlegel RA, Hengartner MO (2007). Aminophospholipid translocase TAT-1 promotes phosphatidylserine exposure during *C. elegans *apoptosis. Curr Biol.

[B24] Chitwood DJ (1999). Biochemistry and function of nematode steroids. Crit Rev Biochem Mol Biol.

[B25] Kurzchalia TV, Ward S (2003). Why do worms need cholesterol?. Nat Cell Biol.

[B26] Merris M, Kraeft J, Tint GS, Lenard J (2004). Long-term effects of sterol depletion in *C. elegans*: sterol content of synchronized wild-type and mutant populations. J Lipid Res.

[B27] Motola DL, Cummins CL, Rottiers V, Sharma KK, Li T, Li Y, Suino-Powell K, Xu HE, Auchus RJ, Antebi A, Mangelsdorf DJ (2006). Identification of ligands for DAF-12 that govern dauer formation and reproduction in *C. elegans*. Cell.

[B28] Rottiers V, Motola DL, Gerisch B, Cummins CL, Nishiwaki K, Mangelsdorf DJ, Antebi A (2006). Hormonal control of *C. elegans *dauer formation and life span by a Rieske-like oxygenase. Dev Cell.

[B29] Matyash V, Geier C, Henske A, Mukherjee S, Hirsh D, Thiele C, Grant B, Maxfield FR, Kurzchalia TV (2001). Distribution and transport of cholesterol in *Caenorhabditis elegans*. Mol Biol Cell.

[B30] Merris M, Wadsworth WG, Khamrai U, Bittman R, Chitwood DJ, Lenard J (2003). Sterol effects and sites of sterol accumulation in *Caenorhabditis elegans*: developmental requirement for 4α-methyl sterols. J Lipid Res.

[B31] Gerisch B, Antebi A (2004). Hormonal signals produced by DAF-9/cytochrome P450 regulate *C. elegans *dauer diapause in response to environmental cues. Development.

[B32] Brenner S (1974). The genetics of *Caenorhabditis elegans*. Genetics.

[B33] Bretscher MS (1973). Membrane structure: some general principles. Science.

[B34] Matyash V, Entchev EV, Mende F, Wilsch-Brauninger M, Thiele C, Schmidt AW, Knolker HJ, Ward S, Kurzchalia TV (2004). Sterol-derived hormone(s) controls entry into diapause in *Caenorhabditis elegans *by consecutive activation of DAF-12 and DAF-16. PLoS Biol.

[B35] van Mil SW, van Oort MM, Berg IE van den, Berger R, Houwen RH, Klomp LW (2004). Fic1 is expressed at apical membranes of different epithelial cells in the digestive tract and is induced in the small intestine during postnatal development of mice. Pediatr Res.

[B36] Eppens EF, van Mil SW, de Vree JM, Mok KS, Juijn JA, Oude Elferink RP, Berger R, Houwen RH, Klomp LW (2001). FIC1, the protein affected in two forms of hereditary cholestasis, is localized in the cholangiocyte and the canalicular membrane of the hepatocyte. J Hepatol.

[B37] Fei W, Alfaro G, Muthusamy B-P, Klaassen Z, Graham TR, Yang H, Beh CT (2008). Genome-wide analysis of sterol-lipid storage and trafficking in *Saccharomyces cerevisiae*. Eukaryot Cell.

[B38] Reiner S, Micolod D, Zellnig G, Schneiter R (2006). A genomewide screen reveals a role of mitochondria in anaerobic uptake of sterols in yeast. Mol Biol Cell.

[B39] Kishimoto T, Yamamoto T, Tanaka K (2005). Defects in structural integrity of ergosterol and Cdc50p-Drs2p putative phospholipid translocase cause accumulation of endocytic membranes, onto which actin patches are assembled in yeast. Mol Biol Cell.

[B40] Paulusma CC, Groen A, Kunne C, Ho-Mok KS, Spijkerboer AL, Rudi de Waart D, Hoek FJ, Vreeling H, Hoeben KA, van Marle J, Pawlikowska L, Bull LN, Hofmann AF, Knisely AS, Oude Elferink RP (2006). Atp8b1 deficiency in mice reduces resistance of the canalicular membrane to hydrophobic bile salts and impairs bile salt transport. Hepatology.

[B41] Nagasaka H, Yorifuji T, Egawa H, Yanai H, Fujisawa T, Kosugiyama K, Matsui A, Hasegawa M, Okada T, Takayanagi M, Chiba H, Kobayashi K (2005). Evaluation of risk for atherosclerosis in Alagille syndrome and progressive familial intrahepatic cholestasis: two congenital cholestatic diseases with different lipoprotein metabolisms. J Pediatr.

[B42] Gengyo-Ando K, Mitani S (2000). Characterization of mutations induced by ethyl methanesulfonate, UV, and trimethylpsoralen in the nematode *Caenorhabditis elegans*. Biochem Biophys Res Com.

[B43] Lyssenko NN, Hanna-Rose W, Schlegel RA (2007). Cognate putative nuclear localization signal effects strong nuclear localization of a GFP reporter and facilitates gene expression studies in *Caenorhabditis elegans*. Biotechniques.

[B44] Maduro M, Pilgrim D (1995). Identification and cloning of *unc-119*, a gene expressed in the *Caenorhabditis elegans *nervous system. Genetics.

[B45] Tucker ML, Whitelaw CA, Lyssenko NN, Nath P (2002). Functional analysis of regulatory elements in the gene promoter for an abscission-specific cellulase from bean and isolation, expression, and binding of three TGA-type basic leucine zipper transcription factors. Plant Physiol.

[B46] Praitis V, Casey E, Collar D, Austin J (2001). Creation of low-copy integrated transgenic lines in *Caenorhabditis elegans*. Genetics.

[B47] Kamath RS, Martinez-Campos M, Zipperlin P, Fraser AG, Ahringer J (2000). Effectiveness of specific RNA-mediated interference through ingested double-stranded RNA in *Caenorhabditis elegans*. Genome Biol.

[B48] Chenna R, Sugawara H, Koike T, Lopez R, Gibson TJ, Higgins DG, Thompson JD (2003). Multiple sequence alignment with the Clustal series of programs. Nucleic Acids Res.

