# Supplementary material for: An unexpectedly high degree of specialization and a widespread involvement in sterol metabolism among the C. elegans putative aminophospholipid translocases
Source: BMC Dev Biol. 2008 Oct 2;8:96. doi: 10.1186/1471-213X-8-96 (PMC2572054; doi:10.1186/1471-213X-8-96)
Supplement: Additional file 1 — WormBase IDs and given names of the six C. elegans ORFs encoding P-type ATPases in subfamily IV. [file 1471-213X-8-96-S1.doc]

**WormBase IDs and given names of the six *C. elegans* ORFs encoding P-type ATPases in subfamily IV.**

| Given name | WormBase ID |
| --- | --- |
| *tat-1*  *tat-2*  *tat-3*  *tat-4*  *tat-5*  *tat-6* | Y49E10.11  H06H21.10  W09D10.2  T24H7.5  F36H2.1  F02C9.3 |
