# Supplementary material for: An unexpectedly high degree of specialization and a widespread involvement in sterol metabolism among the C. elegans putative aminophospholipid translocases
Source: BMC Dev Biol. 2008 Oct 2;8:96. doi: 10.1186/1471-213X-8-96 (PMC2572054; doi:10.1186/1471-213X-8-96)
Supplement: Additional file 2 — Peculiarities of tat-1 and tat-4 transcription. [file 1471-213X-8-96-S2.doc]

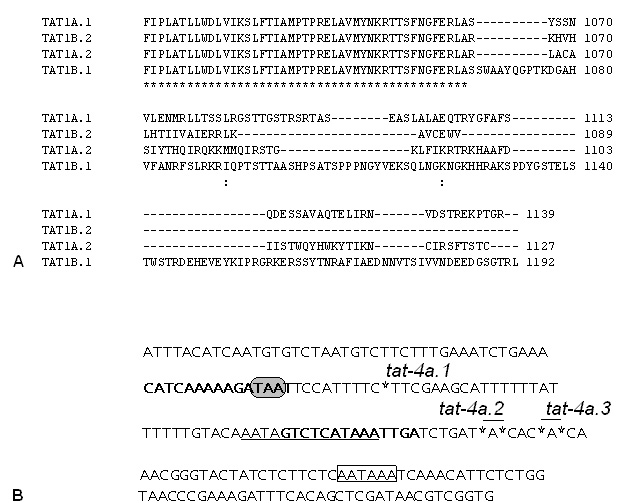


**Peculiarities of *tat-1* and *tat-4* transcription.** (A) The divergent C terminus amino acid sequences in the protein products encoded by the four *tat-1* isoforms. (B) Exon 16 of the *tat-4* ORF. Locations of the poly(A) sites in the three isoforms that terminate in this exon are indicated with a single star or two stars when there is an ambiguous – from either the poly(A) or pre-mRNA – A nucleotide at the poly(A) site. The stop codon is in a shaded oval. Nucleotides in bold and underlined are regions located an appropriate distance away from the putative poly(A) site to serve as poly(A) signals. Curiously, exon 16 contains a canonical poly(A) signal sequence (boxed) that does not seem to function.
