# Supplementary material for: An unexpectedly high degree of specialization and a widespread involvement in sterol metabolism among the C. elegans putative aminophospholipid translocases
Source: BMC Dev Biol. 2008 Oct 2;8:96. doi: 10.1186/1471-213X-8-96 (PMC2572054; doi:10.1186/1471-213X-8-96)
Supplement: Additional file 3 — Fusions of GFP with long N-terminal portions of either TAT-2 or TAT-4 were not expressed due to apparent toxicity. [file 1471-213X-8-96-S3.doc]

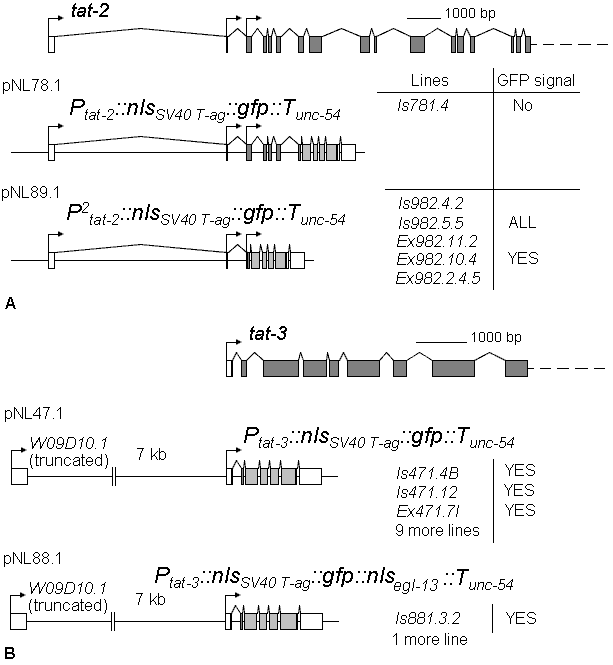


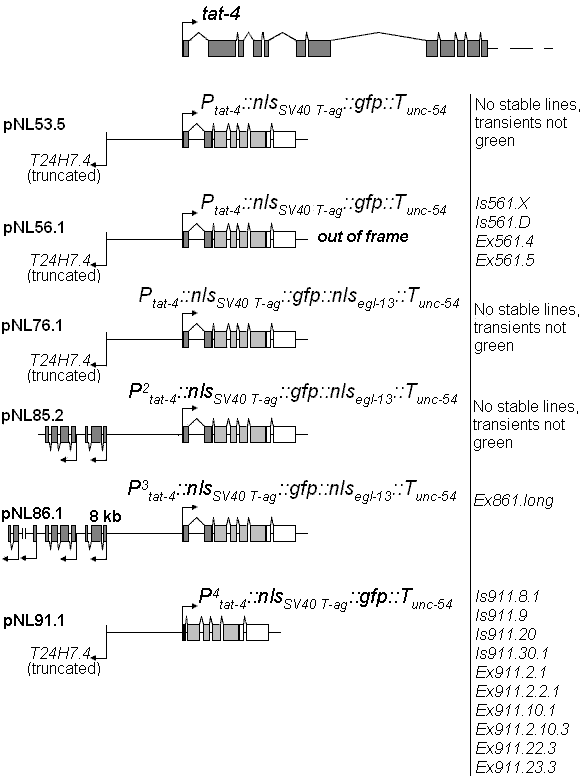
**C**

**Fusions of GFP with long N-terminal portions of either TAT-2 or TAT-4 were not expressed due to apparent toxicity.** (A) Bombardment of pNL78.1, which encodes GFP fused to either 165 (in *tat-2a* and *tat-2b* isoforms) or 200 (in *tat-2c*) amino acids from the N terminus of TAT-2, yielded only one transgenic line, which did not exhibit green fluorescence. In contrast, bombardment of pNL89.1, in which the retained translated portion of *tat-2* was shortened to the region that encodes only either 9 (in *tat-2a* and *tat-2b* isoforms) or 44 (in *tat-2c*) amino acids, yielded many transgenic lines expressing the reporter. (B) The translated portion of *tat-3* included in the *tat-3* expression cassettes codes for only 9 amino acids. Longer translated regions were not tried. (C) A fusion peptide of GFP with 65 N-terminal amino acids of TAT-4 (pNL53.5) could not be expressed. A cassette in which GFP-coding sequence is fused out of frame to the same 65 N-terminal TAT-4 amino acids (pNL56.1) did generate transgenic lines that exhibited tissue-specific green fluorescence. However, expression patterns in the two integrated lines differed from each other and from the expression patter in the two extrachromosomal lines, which suggests that GFP expression in these lines was driven by spurious promoters. Extending the *tat-4* promoter region (pNL85.2 and pNL86.1) included in the expression cassette did not lead to production of the GFP-65aaTAT-4 chimera. But shortening the translated *tat-4* region included in the expression cassette (pNL91.1 codes for only the first 6 TAT-4 amino acids) led to expression of the resultant peptide. Cumulatively, these observations suggest that fusions of GFP with N-terminal regions of TAT-2 or TAT-4 are toxic to nematode cells.
