# Supplementary material for: An unexpectedly high degree of specialization and a widespread involvement in sterol metabolism among the C. elegans putative aminophospholipid translocases
Source: BMC Dev Biol. 2008 Oct 2;8:96. doi: 10.1186/1471-213X-8-96 (PMC2572054; doi:10.1186/1471-213X-8-96)
Supplement: Additional file 4 — Supplementary materials and methods. [file 1471-213X-8-96-S4.doc]

#### Supplementary materials and methods

Gene transcription analysis

Primers used:

| adaptor-dT  GSP_*tat-1_*1  GSP_*tat-2_*1  GSP_*tat-3_*1  GSP_*tat-3_2*  GSP_*tat-4*_1  GSP_*tat-4*_2  GSP_*tat-4*_3  GSP_*tat-4*_4  GSP_*tat-4*_5  GSP_*tat-4*_6  GSP_ *T24H7.6*_1  GSP_*tat-5*_1  GSP_*tat-6*_1  SL1  adaptor-SL1  SL2  adaptor | AAGCAGTGGTATCAACGCAGAGTAC(T)_20_VX, where V is A, G or C and X is any deoxynucleotide  CGCTACCGTAGTCTCTGTAAACAGTC  ATTGAGAGCCTAAAACCGGTGCCTAC  ACTGCTGAGAAGAATGGCCCGTAG  GAAGAAGTTCATCACTAGCCAG  GGAGATGCATCAGGCGCTGGTGAG  TTCAGTAAGTGTTCCTGTTTTGTCTG  CTGAAAGAACATGTGTGACTGTTC  CAGACAAAACAGGAACACTTACTG  ACGACGTTCCAATGATTCAG  AGCCAACGACGTTCCAATG  AGAATGGAAAGGCAGTGTGATGGTCG  ACGGGTGGATTGGTTACTCGCATTAG  AATGTCCCTGTCTTGTCCGAC  GGAATTCGGTTTAATTACCCAAGTTTGAG  AACTGCAGCCGCGGCTCGAGGGTTTAATTACCCAAGTTTGAG  AGAGGTTTTAACCCAGTTACTCAAG  AACTGCAGCCGCGGCTCGAG |
| --- | --- |

A portion of *tat-6* was amplified with primers GSP_*tat-6*_1 and SL1. Gene-specific primers were selected to anneal in the ultimate exon of the ORF based on the data collected by Kohara’s cDNA sequencing project (available through WormBase: www.wormbase.org). Near full-length cDNA transcripts of *tat-1*, *tat-2*, *tat-3*, *T24H7.6* and *tat-5* were amplified using a gene-specific and a splice-leader-(SL)-specific primers:

*tat-1*: amplified near full-length using GSP_*tat-1_*1 and SL1 (SL2 did not produce a product), sequenced 4 clones fully, 4 partially; 3 ESTs sequenced (yk1228h06, yk34c11, yk1496c06); a singe poly(A) signal inferred from the ESTs;

*tat-2*: amplified near full-length using GSP_*tat-2_*1 and SL1 (SL2 did not produce a product), sequenced 3 clones fully, 10 partially at both ends; 1 EST sequenced (yk209d5); a single poly(A) signal inferred from the EST data at WormBase;

*tat-3*: amplified near full-length using GSP_*tat-3_*1 and SL1 (SL2 did not produce a product), sequenced 6 clones fully; amplified 3’ end using GSP_*tat-3_*2 and adaptor-dT primers, sequenced 2 clones; a single poly(A) signal inferred from the 3’ end clones and the EST data at WormBase;

*T24H7.6*: amplified near full-length using GSP_ *T24H7.6_*1 and either SL1 or SL2, sequenced fully 10 clones amplified with SL1 and 2 clones with SL2; a single poly(A) signal inferred from the *tat-4* 3’ RACE data (see bellow) and the ESTs in WormBase;

*tat-5*: amplified near full-length using GSP_ *tat-5_*1 and either SL1 or SL2, sequenced partly 2 clones.

*tat-4* cDNA transcripts could not be amplified in a single round. A nested-PCR approach with several sets of primers was used (see Scheme 1). Primers were chosen to anneal to regions encoding amino acid sequences conserved in all or at least the subfamily IV P-type ATPases. This ensured that if there were transcripts without these particular highly conserved sequences, the protein products of such transcripts would likely lack functionality. GSP_*tat-4*_2, GSP_*tat-4*_3, GSP_*tat-4*_4 are partially overlapping primers that anneal to the sequence encoding the absolutely conserved P-type ATPase DKTGT motif. GSP_*tat-4*_5 and GSP_*tat-4*_6 anneal to the region encoding the ND--MI motif; the ND pair is conserved in nearly all P-type ATPases, and the MI, in all P-type ATPases in subfamily IV.

5’ RACE, primer set 1: adaptor-SL1/GSP_*tat-4*_2 (round one), adaptor/GSP_*tat-4*_3 (round 2), sequenced 3 clones;

5’ RACE, primer set 2: adaptor-SL1/GSP_*tat-4*_1 (round one), adaptor/ GSP_*tat-4*_2 (round 2), sequenced 3 clones;

3’ RACE, primer set 1: GSP_*tat-4*_4/adaptor-dT (round one), GSP_*tat-4*_5/adaptor-dT (round two), sequenced none;

3’ RACE, primer set 2: GSP_*tat-4*_4/adaptor-dT (round one), GSP_*tat-4*_6/adaptor-dT (round two), fully sequenced 9 clones; 3 poly A sites in exon 16 and 1 polyA site in exon 19 inferred from the clones and the EST sequencing data;

3’ sampling: single round amplification using GSP_ *T24H7.6_*1/GSP_ *4_*6 – correct size 1.7kb fragment; yk36b8, yk214f10 and yk126b10 partial 3’ clones sequenced.


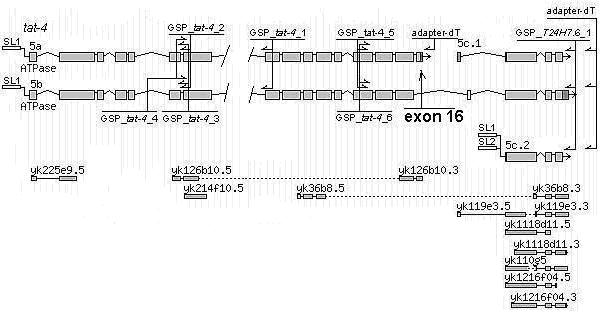


**Scheme 1**: *tat-4* predicted alternative transcripts, available from Kohara’s group *tat-4* cDNA clones and localization of the annealing sites for the primers used to amplify *tat-4* cDNA. (*T24H7.6*  is *5c.1* in the scheme).

Gene expression analysis

*S.* *cerevisiae* cells carrying artificial chromosome Y17G9 were obtained from the *Caenorhabditis* genome sequencing group at the Sanger Institute. A 10ml aliquot of synthetic minimal glucose -ura medium (0.67% Difco™ yeast nitrogen base without amino acids [Difco Laboratories acquired by BD, Franklin Lakes, NJ], 2% dextrose, 0.08% Bio101© Systems CSM -ura w/ 40mg/L ADE [Qbiogene, Irvine, CA]) was inoculated with a large colony of yeast and grown overnight at 30ºC in a rotating wheel. The cell number of the culture was determined using a hemocytometer; the culture was washed twice with cold 50mM EDTA, pH 8: centrifuged at 500g for 5-10min and then re-suspended in 10ml of 50mM EDTA, pH 8 by vortexing. The whole suspension (≥ 2.5 10^8^ cells) was centrifuged again, and the pellet was re-suspended in 600ml of the sorbitol/potassium phosphate/EDTA buffer (1M sorbitol, 50mM potassium phosphate, pH 7.5, 0.02M EDTA, pH 8). This suspension then received 2μl of 2-mercapthoethanol and 50U of zymolyase (Zymo Research via VWR) and was incubated at 30ºC in a rotating wheel (gentle rotations) for 1-2 hours with periodic visual monitoring. Spheroplasts were pelleted by centrifugation in a bucket centrifuge at 3000g for 5-6min; the pellet was re-suspended in 180ml of ATL buffer (DNeasy® kit, Qiagen), and 20μl of proteinase K (DNeasy® kit, Qiagen) was added to the suspension, which was then incubated for 2 hours at 55ºC in a heatblock. Total DNA was isolated from the digestions using a DNeasy® kit (Qiagen, Valencia, CA).

*Escherichia coli* cells carrying cosmids T24H7, W09D10 or F36H2 were obtained from the *Caenorhabditis* genome sequencing group at the Sanger Institute. T24H7 cosmid was unstable in cells grown >30ºC or stored for prolonged (>3 days) periods of time at 4ºC. DNA was extracted from cell cultures with the cosmids using a QIAGEN plasmid midi kit (QIAGEN, Valencia, CA).

The 6.4 kb Swa I/Sal I *tat-5b* promoter fragment that includes two complete upstream ORFs (the first ORF of the operon in which *tat-5* is the second cistron and an independent ORF upstream of the operon) was cloned from the F36H2 cosmid into EcoR I/Sal I cut pBluescript. The promoter was then cut out as Pst I/Sal I segment and ligated into Pst I/Sal I cut pPD95.69 (Fire lab vector kit) to derive pNL45.3, in which the translated portion of *tat-5b* is out of frame with the translated reporter sequence. To bring the two into the same frame the Xba I site was destroyed in pNL45.3 to derive pNL48.1 (Figure 3A).

A 4.7 kb *tat-5a* promoter fragment was obtained from F36H2 in two portions as the Sal I/Nco I restriction segment and as a PCR fragment amplified with primers 4188 (TCGACGTCTTCGTCCACATTTAG) and 4189 (GGATCCTACTCGAACCGTTCGTGAG). The PCR product was sequenced. The two portions were assembled into pPD95.69 to derive pNL46.1 (Figure 3A). pNL48.1 and pNL46.1 were used in particle bombardment.

A 7.6 kb segment that includes 2 introns of the *tat-2* ORF and a 1.1 kb proximal promoter region was amplified from Y17G9 in three overlapping fragments using proof-reading polymerase PrimeSTAR™ (Takara Mirus Bio, Madison, WI) with primer pairs 9060 (GCGGATCCTGAACAGCATGGCAACCAACTG) and 7154 (TGTACTTTTCCCCTCTTGTGAAC), 7153 (CATTTTATGCAGAGTTCGAGTCAC) and 7152 (GAAGAGTGGCGGTAAAATTCGG), and 7151 (TCTTCTAACTCTCGGGCCATTTG) and 7150 (TAACATCCAAAAGCAGTAACATCC). The 2.2 kb fragment amplified with 9060 and 7154 primers was sequenced. The three fragments were then assembled together into pPD95.69 (Fire lab vector kit) to derive pNL89.1 (Figure 4A).

The *tat-3* promoter sequence (the 7 kb segment between *tat-3* and the preceding ORF, plus the first exon-intron pair) was obtained from the W09D10 cosmid in two overlapping fragments: as a distal 5.6 kb Spe I/Sph I restriction digest fragment and as a proximal 1.8 kb PCR fragment amplified with primers 4282 (CCAAATCCCAACTTTCTGCCA) and 4281 (GACGTCGGATCCTTCCACGTCCACCGACTTCTG). The PCR amplified segment was sequenced. The two fragments were reassembled into pPD95.69 (Fire lab kit) to derive pNL47.1, and then the full promoter was cut out from pNL47.1 as the Pst I/Nco I fragment and inserted into Pst I/Nco I cut pNL74.4 (a derivative of pPD95.69 in which a second NLS from EGL-13 was added to the C terminus of GFP) to derive pNL88.1. pNL47.1 and pNL88.1 were used for particle bombardment (Figure 5A).

A *tat-4* expression cassette was assembled by amplifying a 1.6 kb *tat-4* promoter fragment with primers M13-rev (standard primer) and 9059 (GCGGATCCTGGTCGAGGTGTCGTCATATTC) from pNL53.5 and then replacing the longer Nhe I/BamH III fragment in pNL53.5 with the shorter Nhe I/BamH III fragment from the PCR product to derive pNL91.1 (Figure 6A). pNL53.5 was made by inserting into Pst I/BamH III cut pPD95.69 (Fire lab vector kit) a Pst I/BamH III segment amplified with primers 5583 (CTGCAGCGATATGTCATCAACTCCGGTC) and 5584 (GGATCCGTCAGTGAAGTCTCGGTAGTTG) from the T27H7 cosmid. Amplified regions in pNL53.5 and then again in pNL91.1 were sequenced.

*tat* expression cassette vector DNA and DNA of the vector carrying *unc-119(+)* (pDP#MM016B) were isolated from *E. coli* cultures using the QIAprep Spin Miniprep kit (Qiagen, Valencia, CA). 4.5μg of an expression vector were combined with 1.5μg of the *unc-119(+)* vector for the total of 6μg of DNA (in the 3:1 w/w ratio) used to coat 3mg of gold particles.

Around 3mg of gold particles (in almost all cases 1μm in diameter; in the remaining cases 1.6μm gold particles were used) (Bio-Rad Laboratories, Hercules, CA) were placed into a high quality siliconized 1.5ml microcentrifuge tube (Bio Plas, San Rafael, CA). The particles were then washed three times with alcohol and once with distilled water: washed with 200μl of either alcohol or water by vigorous vortexing for 1min (very brief vortexing with water), briefly (<10sec with alcohol and slightly longer with water) centrifuged at no more than 100 g in a standard tabletop centrifuge, liquid removed without loss of the gold particles. After washing, the gold particles were re-suspended in the amount of water that was determined by the formula: 55 – the number of μls of the DNA solution containing 6μg of vector DNA = μl amount of water for gold particle re-suspension (the total DNA solution plus water volume did not exceed 55μl). Following re-suspension, the gold particles, while being vortexed, were quickly combined in the following order with the DNA solution, 50μl of 2.5M CaCl_2_ and 20μl of 0.1M spermidine and then vigorously vortexed for 3 more minutes. The coated particles were pelleted to remove the supernatant, washed once with alcohol (as above) and re-suspended by vigorous vortexing in 100μl of alcohol. The suspension was sonicated for 5-10sec in a water-bath sonicator and then immediately spotted in 10μl aliquots onto macrocarriers (due to evaporation of alcohol there was enough suspension to spot 8-9 macrocarriers).

*unc-119(-)* nematodes were grown on 14cm enriched peptone plates spotted with *E. coli* bacteria of either C600 (a kind gift of Dr. C Malone) or OP50 strain. Before bombardment nematode plates were passaged (animals washed off with M9 buffer to one side of the plate held under incline and then transferred by pipetting to new plates) 1 to 2 or 3 every day for 2 or 3 days. Even with frequent passages, plates always contained significant areas in which nematodes cleared all food and collected into heaps. The heaped animals were in most cases spread out with a bent and moistened Pasteur pipette immediately before bombardment.

Particle bombardment was conducted using a PDS-1000/He system (Bio-Rad Laboratories, Hercules, CA) as recommended by the manufacturer. In almost all cases 1350psi rupture disks were used. All other parameters were standard presets used for plant bombardment [44]. Each nematode plate was bombarded in most cases only once.

After bombardment, most nematodes were washed off a bombarded plate with M9 buffer and transferred to 2 or 3 fresh plates. The original and the new plates were incubated under standard conditions for 1 to 3 weeks before screening for transgenic animals. For early screening (only 1 week incubation), plates were flooded with M9 buffer and then all animals that swam were collected onto fresh plates. Some of these animals were only transiently transgenenic and produced exclusively uncoordinated (*unc-119*) progeny. Only one stably transgenenic line, considered independent, was selected per screened plate. Integrated lines were identified by whether homozygous for the transgene animals (not producing *unc-119* progeny) could be derived. Stably transgenic lines, either integrated or extrachromosomal, were maintained under regular conditions for many generations without loss of the transgene.

RNA interference

Partial cDNA clones (expressed sequence tags, ESTs) yk23e10, yk34c11, yk126b10, yk209d5 and yk395e5 were kindly provided by Dr. Kohara. Plasmid pPD129.36 contains a multiple cloning site in between two inverted T7 promoters. 800bp Spe I/Hind III fragment of yk34c11 was fused into Spe I/Hind III cut pPD129.36 to derive pNL16.1. 1.8kb Xho I/Not I fragment of yk209d5 was fused into Xho I/Not I cut pPD129.36 to derive pNL20.10; the latter plasmid was then cut with EcoR I and ligated on itself to derive pNL21.1, which harbors only 500bp of the yk209d5 sequence. 1.1kb Bgl II/Pst I fragment of yk395e5 was fused into Bgl II/Pst I cut pPD129.36 to make pNL19.4. 900bp Xba I fragment yk126b10 was fused into Xba I cut pPD129.36 to make pNL18.8. And finally, 700bp Spe I/Hpa I fragment of yk23e10 was ligated into Spe I/Sma I cut pPD129.36 to obtain pNL10.1. Plasmids pNL16.1 (*tat-1*), pNL21.1 (*tat-2*), pNL19.4 (*tat-3*), pNL18.8 (*tat-4*) and pNL10.1(*tat-5*) were used for production of dsRNA for RNAi with the targeted genes.

pNL16.1, pNL21.1, pNL19.4, pNL18.8, pNL10.1 and pPD129.36 (mock control) plasmids were transformed into bacteria of HT115(DE3) *E. coli* strain. Bacterial cultures carrying one of the plasmids each were grown to OD_595_=0.4 with shaking at 37ºC in regular Luria Broth medium enriched with ampicillin to 75μg/ml; IPTG (isopropyl *β*-D-1-thiogalactopyranoside) was then added to 0.4mM to induce transcription of the gene fragments, and the cultures were incubated at the same conditions for 2 more hours. Subsequently, the cultures were centrifuged to remove the supernatant and then re-suspended in a half of the original volume of fresh LB plus 75μg/ml ampicillin and 0.4mM IPTG. Aliquots of the suspensions were spotted onto regular NGM plates and the plates incubated overnight at room temperature. Synchronized newly hatched in the absence of food L1 larvae were placed 20-30 per *tat* or mock dsRNA plate. The nematodes were first observed for any induced phenotypes 3 days later.
